# Clinical and Preclinical Cognitive Function Improvement after Oral Treatment of a Botanical Composition Composed of Extracts from* Scutellaria baicalensis* and* Acacia catechu*

**DOI:** 10.1155/2016/7240802

**Published:** 2016-11-30

**Authors:** Mesfin Yimam, Bruce P. Burnett, Lidia Brownell, Qi Jia

**Affiliations:** ^1^Unigen, Inc., 3005 1st Ave., Seattle, WA 98121, USA; ^2^Entera Health, Inc., 2000 Regency Parkway, Ste. 200, Cary, NC 27518, USA

## Abstract

Dementia and cognitive impairment have become the major concerns worldwide due to a significantly aging population, increasing life span and lack of effective pharmacotherapy. In light of limited pharmaceutical drug choices and the socioeconomic implications of these conditions, the search for safe and effective alternatives from natural sources has gained many attractions within the medical food and dietary supplement industry. Two polyphenol extracts derived from roots of* Scutellaria baicalensis* and heartwoods of* Acacia catechu* containing free-B-ring flavonoids and flavans, respectively, were combined into a proprietary blend called UP326. A similar bioflavonoid composition, UP446, has been reported with modulation of pathways related to systemic inflammation. To test the effect of UP326 on memory and learning, a radial arm water maze (RAWM) and contextual fear conditioning (CF) were utilized in aged F344 rats fed with UP326 at doses of 3, 7, and 34 mg/kg for 11 weeks. The 7 and 34 mg/kg dosage groups had significantly fewer errors than aged vehicle control animals and their performance was equivalent to young animal controls. In a separate human clinical trial, test subjects orally given 300 mg of UP326 BID for 30 days showed marked improvement in speed and accuracy of processing complex information in computer tasks and reduced their standard deviation of performance compared to baseline and the placebo group. This data suggest that UP326 may help maintain memory, sustain speed of processing, and reduce the number or memory errors as we age.

## 1. Introduction

There is a growing interest in alternative medicine and use of dietary or nutritional supplements that have a variety of putative beneficial activities, including weight loss, athletic performance, general health, and cognition. Of particular interest are traditional homeopathic products that can stabilize or enhance cognitive function. Among these conditions, Alzheimer's, disease (AD), a slow neurodegenerative disorder of the brain characterized by progressive cognitive dysfunction and memory impairment, is a major public health concern [1, 2].

There is no cure for AD. The pathophysiology of AD is due to a combination of factors including systemic inflammation [3], oxidative stress [4], and beta-amyloid plaque formation [5]. Aging and oxidative stress are associated with declines in hippocampal processing of information [6–8] as demonstrated by the deficits seen in spatial learning, memory formation, and the decline in long term potentiation that is necessary for memory consolidation. Multiple reports have identified a correlation between COX-2 expression, reduction in eicosanoid production, systemic inflammation (i.e., TNF*α*, MIP1-*α*, and IL-1*β* expression), and the pathogenesis of AD leading to loss in memory and speed of information processing in specific animal models [9, 10]. The protective effect of nonspecific anti-inflammatory drugs (NSAIDs) in the pathogenesis of AD is attributed to COX-2 inhibition and the direct prevention of amyloid plaque accumulation in the brain. By suppressing COX-2 production and hence reducing the proinflammatory prostaglandin PGE_2_, the surrounding neurons will be spared from the oxidative stress and inflammatory insult that would be generated by activated microglia [10]. This action eliminates the subsequent microglial generation of cytokines and reactive oxygen species (ROS) that feed the cycle and propagate the neurodegeneration [11, 12]. By doing so, NSAIDs may impact the incidence of diminished cognitive function in the elderly as long as treatment is administered decades earlier [13]. However, in spite of numerous beneficial usages of NSAIDs, the chronic administration of these drugs is limited due to the adverse effects on gastrointestinal, renal, and cardiovascular systems. Dietary supplements could potentially fulfill the desired objectives in minimizing the chances of later age memory loss without compromising the safety of the patient.

Dietary supplements enriched in polyphenols, individually and in combination, may provide substantial benefits including neuroprotection from oxidative and inflammatory insults and improve neuronal functions. In this regard, flavonoids from* Scutellaria baicalensis* have been found to attenuate learning and memory impairment and improve cognitive function in Alzheimer's disease models. For example, these flavonoids have been found to improve spatial learning and memory in VaD rats which have a bilateral block of the common carotid artery that results in chronic cerebral ischemia resulting in vascular dementia [14]. When Sprague-Dawley-derived cortical cells were exposed to cyanide, inclusion of* Scutellaria* flavonoids attenuated oxidative stress-induced apoptosis of cortical neurons [15]. These antioxidant effects of* Scutellaria* flavonoids may also specifically prevent neuronal loss induced by amyloid beta-peptide. When Wistar rats were prefed with* Scutellaria baicalensis* stem-leaf total flavonoid preparations and then amyloid beta-peptide injected into the bilateral hippocampus, resulting apoptosis was attenuated compared to control animals [16]. Baicalin also provided neuroprotection against global ischemia/reperfusion injury via activation of GABAergic signaling [17], inhibited inflammatory activation of microglia [18], protected against glutamate/NMDA excitotoxicity [19], mitigated okadaic acid-induced neuronal damage [20], protected against oxidative stress-induced DNA damage by upregulating the DNA repair system [21], and reduced hippocampal neuronal damage in global cerebral ischemia model through the inhibition of MMP-9 activity [22]. The result of baicalin neuroprotection was also observed as improved cognitive function in senescent mice [23]. Likewise, catechin from* Acacia catechu* has also showed antiamnesic [24] and antineurodegenerative [25] properties suggestive of its usage in alleviating cognitive disorders. The combination of baicalin and catechins from* Scutellaria baicalensis* and* Acacia catechu* has been reported with regulatory proinflammatory pathways by inhibiting COX/LOX and downregulating cytokines [26, 27].

Given the above implications and significance, the application of a supplement with anti-inflammatory, antioxidative, and a wide range of neuroprotective activities will certainly have a merit of use in relieving memory impairment. Although* Scutellaria baicalensis* and* Acacia catechu* can potentially support neural functions, it is not yet established if they can impact learning and memory. The present study was therefore designed to analyze the effects of UP326, a composition containing a proprietary blend of two standardized extracts consisting primarily of baicalin from* Scutellaria baicalensis* Georgi (Family: Lamiaceae) (Chinese skullcap) and (+)-catechin from* Acacia catechu* (Family: Mimosaceae) (black catechu), on short term memory in aged rats. A preliminary pilot human clinical study was also carried out to assess the impact of the composition on speed and accuracy of processing complex information.

## 2. Materials and Methods

### 2.1. The Composition

**Figure 1:**
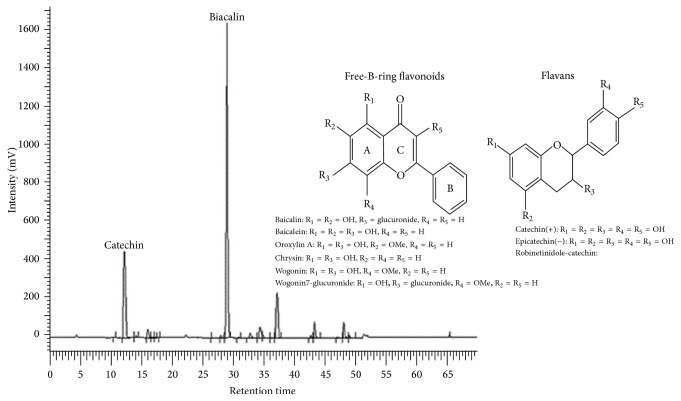
HPLC chromatogram and chemical structure of free-B-ring flavonoid (baicalin) and flavan (catechin). The flavonoids were detected using a UV detector at 275 nm and identified based on retention time by comparison with known flavonoid standards.

Detailed methods for preparing extracts enriched for baicalin and catechin from the roots of* Scutellaria baicalensis* and the heartwoods of* Acacia catechu*, respectively, were disclosed in a USA patent [28]. UP326 is the combination of a standardized baicalin extract from* Scutellaria* and a standardized catechin extract from* Acacia* with baicalin content of not less than 60% and catechin content of not less than 10%. Extracts and UP326 were analyzed by high-performance liquid chromatography (HPLC) using a Phenomenex Luna 5 *μ*m C-18, 250 mm × 4.6 mm with a C-18 Security Guard cartridge in a column oven at 35°C. The mobile phase had a flow rate of 1.0 mL/min and used an isocratic 1% phosphoric acid : acetonitrile ratio of 85% : 15% for the first 7 min and then a new gradient to 10% : 90% from 7 to 16.5 min and then an isocratic 1% phosphoric acid : acetonitrile gradient with a ratio of 85% : 15% for 7.5 min. The flavonoids were detected using a UV detector at 275 nm and identified based on retention time by comparison with known standards (Figure 1).

### 2.2. Preclinical Study

#### 2.2.1. Animals

Fischer 344 (F344) rats (young, 6 months of age, *n* = 44; and aged, 17 months of age, *n* = 56) were purchased from Harlan Sprague Dawley. Fisher 344 (F344) rats are general multipurpose model most frequently used for aging, safety, and efficacy testing. Rats were fed with pellet diets of either NIH31 (control) or NIH31 + UP326 (3, 7, or 34 mg/kg). There were two separate studies conducted. Experiment A was an aging study that examined the effects of UP326 on age-related declines in learning and memory. There were 12 young (6-month-old) rats and 48 aged (17-month-old) rats. Due to the large number of animals, the experiment was split into two cohorts of 30 rats, with each group containing 6 animals. The animals were first assessed in the radial arm water maze (RAWM) task prior to being placed on the experimental diet [29, 30]. Upon completion of the initial RAWM test, the aged rats were assigned to one of the four groups (aged control, 3, 7, and 34 mg/kg UP326) in a counterbalanced manner such that each group was equivocal in RAWM performance. Experiment B assessed the anxiolytic effects of UP326 on anxiety using elevated plus maze and open field studies in a group of young F344 rats (*n* = 32). The animals were randomly assigned to one of the four groups (control, 3, 7, or 34 mg/kg UP326).

#### 2.2.2. Radial Arm Water Maze (RAWM)

The RAWM consisted of 12 arms (15 cm wide × 43 cm long) emanating from a circular choice area (60 cm diameter) in a 1.5 m tank of water. An escape platform (10 cm × 13 cm) was situated at the end of one of the arms, 2 cm below the surface of the water. Rats were pretrained in the maze for 5 days. Pretraining consisted of shaping the animals to find the goal arm by initially blocking entry into the nongoal arms and gradually increasing the number of available arms until all 12 were open. The rats were then subjected to the main study conducted at two blocks of 5 days each for a total of 10 days. During the main study, the start arm for each trial was determined in a random manner from the 11 available arms. To avoid place and position preferences, the start and goal arms were different for each animal within a group on a given day but equivalent across groups. Four trials were administered per day (180 s maximum) with a 30 s intertrial interval. If a rat did not find the escape platform within 180 s, it was gently guided to the correct arm. Errors, that is, the number of arms entered prior to entering the arm containing the escape platform, were recorded for each rat. A 3 h delay was introduced between trials 3 and 4 for days 6 through 10. During the delay, the rats were placed back into their home cage. Data are presented as the means for each trial across days.

#### 2.2.3. Contextual Fear Conditioning

One week after completing the RAWM training, the rats were placed in a box (30.5 cm × 24.1 cm × 21 cm, Med Associates, St. Albans, VT) with a grid floor (4.8 mm diameter rods, spaced 1.6 cm apart) connected to a constant current shocker (Med Associates). Prior to placing each rat in the box, the box was cleaned with 3% acetic acid, which functioned as a specific odorant for the original context. Two consecutive training blocks were administered. Each training block was 180 s long with a 30 s, 85 dB white noise conditioned stimulus and a 2 s, 0.5 mA foot shock. The conditioned stimulus and foot shock were coterminated at the end of the training block. All rats reacted to the foot shock by jumping. The rats remained in the training box for 30 s following the second training block. Retention was tested 2 days after training by first placing the animals in the same apparatus, using 3% acetic acid as an odorant, in which training was performed for 5 min, without the conditioned stimulus or foot shock. Two to three hours later, the rats were placed in the same chamber except that the grid floor was covered with a piece of black formica and the cage was cleaned with 3% ammonium hydroxide (novel context) for 6 min, during which the conditioned stimulus was administered for the final 3 min. Freezing was quantified manually every 10 s by a researcher blind to the treatment groups of the rats. At 10 s intervals, the researcher assessed whether the rat was freezing or not [31–33]. Percent freezing was calculated as the number of intervals during which the rat was assessed as freezing/the total number of intervals × 100.

### 2.3. Clinical Study

#### 2.3.1. Subjects

Health adults (*n* = 83), 35 to 65 years of age, recruited though the internet participated in the study. Subjects were randomly assigned to placebo (*n* = 40) and treatment (*n* = 43) arms, with groups balanced for age and gender. Group A received inactive placebo; group B received UP326 with a dosage of 300 mg/day, a proprietary combination of two herbal extracts,* Scutellaria baicalensis* and* Acacia catechu, *for a duration of 30 days. Study was conducted at CRO: Cognitive Care, Inc., University of California-Irvine, Department of Neuroscience (CA, USA). Protocol, informed consent, advertisement, and other trail related materials were reviewed by ESSEX Institutional Review Board, Inc., when applicable.

#### 2.3.2. Behavioral Assessments

A battery of cognitive tests was administered each week over a 4-week treatment period to assess the influence of the study composition. Before the study start, participants were required to practice the tests on two consecutive days. Baseline performance was established by testing before treatment dosing. The data analysis compares baseline performance and treatment conditions to determine the impact of the treatment conditions on cognitive function. Measures of cognitive performance were obtained using web-based assessments from the Cognitive Care battery tests of Working Memory Speed (executive decision-making, quickness, and flexibility).

*Working Memory Speed* presents a word and picture simultaneously and requires a decision to state if they are the same or different. A reversal cue is also presented randomly and requires the person to respond opposite of the correct response, so that a response to a correct pair would be no and* visa versa*. This task requires suppression or “inhibition of a learned response” and then a reversal (“task shifting”) of the response contingency. This speed of switching from one task or one response mode to another is often equated with mental flexibility and higher-order cognitive processing, as well as superior decision-making [34–38].

### 2.4. Statistical Analysis

The analyses are 2-way ANOVA with repeated measures. If there was a significant effect of group and a significant group × trial interaction (preclinical) or group × week interaction (clinical), then* post hoc* analyses were performed using the Bonferroni/Dunn correction for multiple comparisons. An uncorrected *p* value of less than 0.05 was considered statistically significant.

## 3. Result

### 3.1. Preclinical Study

#### 3.1.1. RAWM

**Figure 2:**
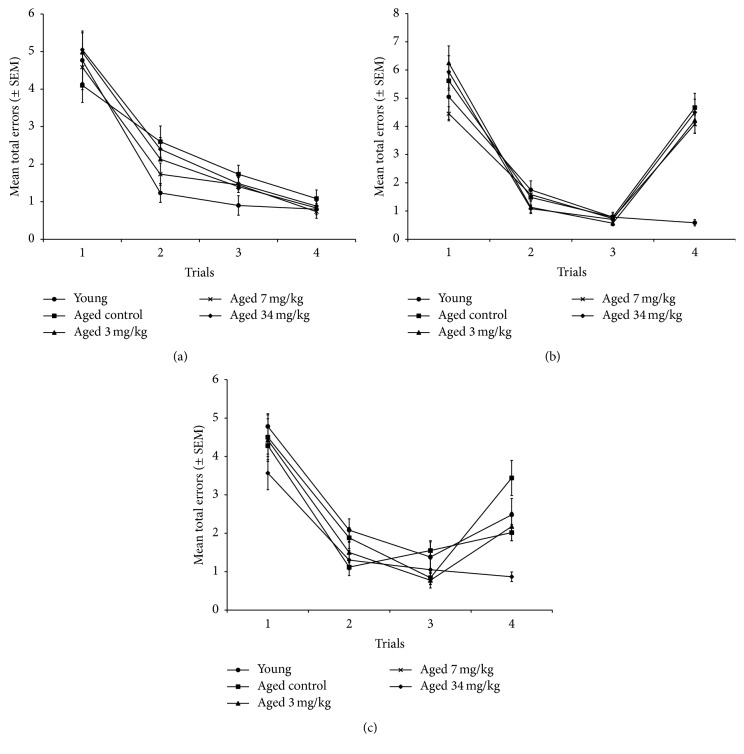
Radial arm water maze (RAWM) study. After initial training in the RAWM but before administration of UP326, aged rats were able to perform as well as their young cohorts (Figure 2(a)). But after a 3 hr delay was inserted between trials 3 and 4, the aged rats lost all training advantages (Figure 2(b)). After taking UP326 for 2 months, all dose groups showed significant improvement attenuation of age-related memory impairments (Figure 2(c)). 12 rats per group were used for each study.

During the baseline training, all animals learned the basic underlying principles of the task as evidenced by the rapid decrease in the number of total errors across trials (Figure 2(a)). There were no group differences [*F*(4,55) = 1.203, ns] during the initial RAWM testing without delay. When a 4 h delay was inserted between trials 3 and 4, there was a clear effect of age [*F*(4,55) = 4.905, *p* < 0.002] (Figure 2(b)).* Post hoc* comparisons using the Bonferroni/Dunn adjustment for multiple comparisons revealed that all the aged groups had significantly more total errors than the young group (*p*'s < 0.0001). Following the baseline training, the aged animals were assigned to treatment groups (control, 3, 7, and 34 mg/kg UP326) in a counterbalanced manner based on their performance on trial 4 of the 4 h delay baseline test. After 11 weeks of treatment, the rats were tested again on the 4 h delay version of the task (Figure 2(c)). There was a significant effect of trial [*F*(3,141) = 103.6, *p* < 0.0001]. All groups decreased the number of errors from trials 1–3. There was significant group effect on trial 4. The aged controls had significantly more errors on trial 4 than the young controls. The UP326-treated animals were not significantly different from young controls. Two of the UP326-treated groups (3 and 34 mg/kg) had significantly fewer errors than the aged controls (*p* < 0.005).

#### 3.1.2. Fear Conditioning

**Figure 3:**
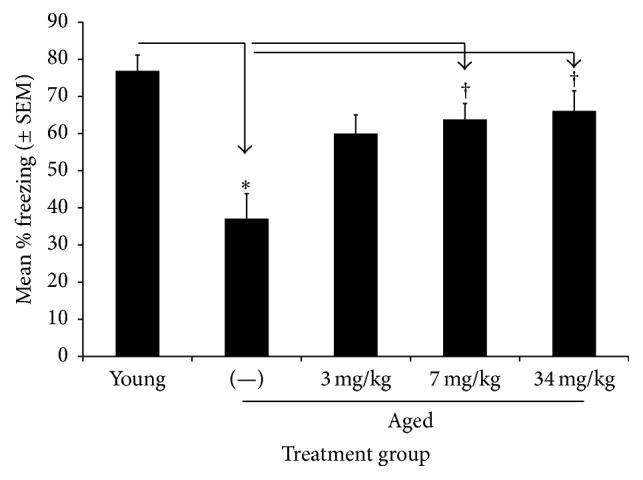
Effects of UP326 on behavior and memory were also tested using contextual fear conditioning (normal freezing behavior to an electrical shock). Contextual fear testing showed an improvement in normal freezing behavior in aged rats given UP326 approaching that of the young control rats. There was no effect of age or UP326 treatment to freezing to either the auditory cue or novel context. *∗* = *p* < 0.0001 versus young controls; ^†^ *p* < 0.0005 versus aged controls. 12 rats per group were used for each study.

There was a significant effect of treatment group on freezing to the original training context [*F*(4,45) = 7.941, *p* < 0.001]. The aged control group froze significantly less than the young group (*p* < 0.0001). The 7 and 34 mg/kg UP326-treated groups froze significantly higher than the aged controls (*p*'s < 0.0005) (Figure 3).

Elevated plus maze and open filed studies were also carried out to assess the anxiolytic effect of UP326 on young (6-month-old) animals. No behavioral changes were observed as a result of UP326 for all the dosages (data not shown).

### 3.2. Clinical Study

#### 3.2.1. Adverse Events

All study compounds were well tolerated with no reports of serious or unexpected treatment related adverse effects. All subjects completed the study.

#### 3.2.2. Working Memory Speed

**Figure 4:**
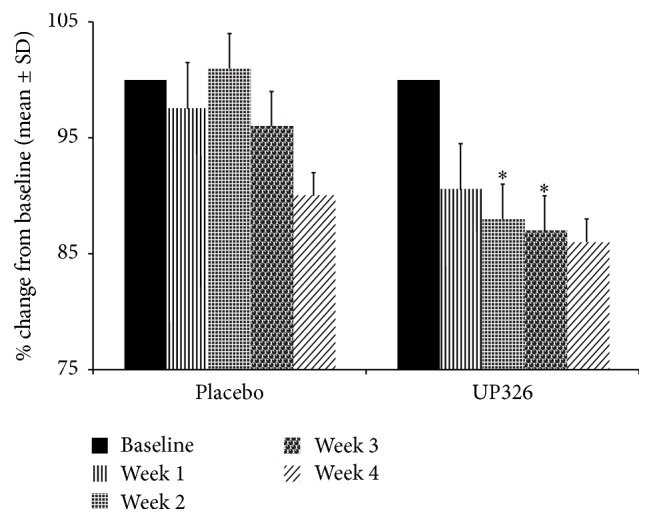
Working memory speed RT median showed improvement from baseline to week 4. Relative to group A placebo, a significant improvement in working memory processing efficiency seen for groups B (UP326) in weeks 2 and 3 of treatment (^*∗*^ *p* < 0.05).

The reaction time median (RT med) scores, a measure of executive function, show the most promising results for this study (Figure 4). The main group effect did not indicate significant differences between groups (*p* = 0.1314); however, there were significant improvement across weeks of testing (*p* < 0.0001) and a significant group × week interaction (*p* = 0.05). Across the weeks of testing, there was a decrease in the RT median from baseline to week 4, indicating a faster rate of responding. For the week by group interaction, subjects in group B showed the largest improvement (e.g., reduced RT median for more rapid performance) (*p* < 0.05) during the weeks 2 and 3 of treatment. These results suggest that the composition can increase cognitive processing (decision-making) speed without impairing choice accuracy, improving the rate of responding to cognitively demanding or complex choice situations.

#### 3.2.3. Reaction Standard Deviation (RTSD)

**Figure 5:**
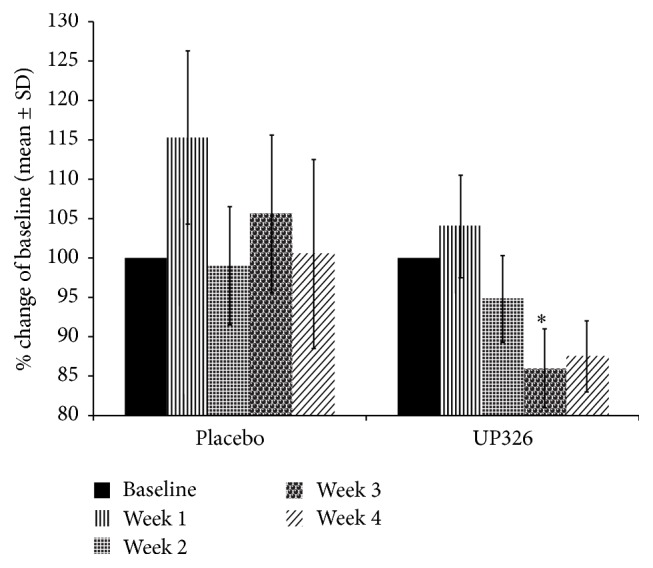
Working memory speed and reaction time standard deviation showed improvement from baseline to week 4. Relative to group A (placebo), a significant improvement in working memory processing efficiency seen for groups B (UP326) in week 3 of treatment (^*∗*^ *p* < 0.05).

 Reaction standard deviation (RTSD) also suggests some promising findings complementing the working memory speed (Figure 5). RTSD is often used as a measure of attention and in the cognitive sciences is typically considered to reflect processing efficiency and neural noise [38]. There is no significant difference between groups (*p* = 0.3962); however, there was significant improvement across weeks of testing (*p* < 0.0002) but there was no significant group × week interaction (*p* = 0.303). Across the weeks of testing, there was a decrease in the standard deviation from baseline to week 4. For the weekly between-group differences, the significant improvement (e.g., reduced standard deviation for more consistent performance) was for group B at week 3 of treatment. These results suggest that the composition may increase sustained attention, improving the consistency of responding to cognitive demanding or complex choice situations.

## 4. Discussion

Due to significantly aging population, increasing life span, and lack of effective pharmacotherapy options, AD and vascular dementia have become the major concerns worldwide. In spite of advances in therapeutic discoveries, there is no cure for AD. Significant bodies of evidence have been documented detailing the pathogenesis of AD in relation to inflammation [3], oxidative stress [4], and beta-amyloid plaque formations [5]. In this report, we have assessed the effect UP326, a composition containing a proprietary blend of two standardized extracts from* Scutellaria baicalensis* and* Acacia catechu* for short term memory in aged rats and for speed and accuracy of processing complex information in humans.

While the nonsteroidal anti-inflammatory drugs seem to possess activities suggestive of benefit in neurodegenerative diseases, their chronic consumption is hindered due to their gastrointestinal, renal, and cardiovascular side effects. To achieve a meaningful therapeutic effect, patients with AD or dementia have to be in a long term treatment schedule. This condition will expose the patients to deleterious pharmacotherapy adverse effects. Hence, there is always a need for safe and efficacious options from natural sources to help maintain cognitive health. Besides its beneficial activities described here, rigorous safety evaluations have been carried out on the composition, UP326,* in vitro* and* in vivo*. For example, 26-week repeated oral toxicity study in rats [39] and in beagle dogs [40], reproductive toxicity studies in rabbits and rats [41–43], a 90-day repeated oral toxicity study in rats [44], and toxicity studies in mice [45] have been performed. The composition also, when dosed in Fischer 344 rats, a model for gastric toxicity of NSAIDs, showed no evidence of ulceration [45].* In vitro*, no mutagenicity or drug interactions were seen by AMES and cytochrome P450 enzyme inhibition, respectively [45]. Data from all these studies attest the safety of UP326 for long term consumption.

The overall strength of the preclinical* in vivo* experiments found in the current study is that UP326 reversed age-related memory deficits in rats in two different learning and memory tasks. In the experimental design, studies conducted at the no delay condition demonstrate the animals' ability to perform the task and act as a control for differences in the ability to perform the task (e.g., locomotion, vision, and motivation). The delay task introduces a 4 h delay between trials 3 and 4, making the task more difficult. It is under the delay condition that the age-related memory impairments were demonstrated. Treatment of aged F344 rats with middle and high doses of UP326 reversed an age-related impairment in spatial memory 11 weeks after treatment. In the contextual fear conditioning task, there was a significant age-related impairment in hippocampal-dependent memory, as demonstrated by the decreased freezing in the training context. This age-related memory impairment was ameliorated by treatment with middle and high doses of UP326.

In the pilot clinical study, performance for the working memory task was already high at baseline in this healthy population. The lack of significance between group differences for working memory speed performance and flexibility may be due to the study participants having normal cognitive function that is close to their maximal level of ability. Implying that ceiling effects were attained, this leaves no room for treatment related statistically significant improvements. Regardless, results from this study suggest that the composition may increase sustained attention and improve the consistency of responding to cognitive demanding or complex choice situations. The data also further warrant additional studies involving large numbers of participants targeting the right age group.

Previously, UP326 has been reported to have a high ORAC value of 70,930 *μ*mole TE/g suggesting its capacity to reduce ROS [46]. When inflammation was induced by 10 ng/mL LPS in PBMCs and treated with UP326, average gene expression was decreased 63-fold for* cox-2*, 45-fold for* il-1β*, 3.3-fold for* tnf-α*, 37-fold for* il-6*, and 2.2-fold for* nfκb* [46]. The 50% inhibitory concentration for inhibition of both COX-1 and COX-2 peroxidase enzyme activities was 15 *μ*g/mL, while the mixed extract showed a value for 5-LOX enzyme activity of 25 *μ*g/mL [27, 45]. Clearly, it is evident from these reports that the standardized composition has intriguing anti-inflammatory and antioxidative activities suggestive of its application in helping to maintain neurocognitive function in healthy populations.

Furthermore, augmented reports have been documented from various animal models describing the beneficial usage of flavonoids in attenuating the characteristic symptoms of AD. In particular, active components such as baicalin, wogonin, and Oroxylin A from* Scutellaria baicalensis* and catechins from* Acacia catechu* extracts have demonstrated their cognitive deficit amelioration effect in multiple* in vivo* models. It has been postulated that these effects could be as a result of their anti-inflammatory and free radical scavenging activities. For instance, baicalein administered orally ameliorated memory impairments induced by chronic bilateral common carotid artery occlusion used as a model for vascular dementia [47] and *β*-amyloid peptide infusion induced model for amnesia [48]. In addition, wogonin also provided neuroprotective effects in cerebral ischemic injury, where these effects have been shown to be mediated by its anti-inflammatory properties via suppression of microglial activation through the inhibition of NF-kB activity [49, 50]. In fact, there is inflammation centered neurodegenerative disease hypothesis for AD based on activation of the microglia as the driving force for neuroinflammation, where redox dysregulation leading to inflammatory gene expression is the common denominator [6]. Similar improvements in memory impairments were also observed for Oroxylin A when administered to mice in intransient cerebral hypoperfusion [51] and scopolamine induced disease models [52] suggesting that improvements may be due to inhibiting cytokine production via reduced oxidative stress in the brain. Similarly, the main active component of* Acacia catechu*, catechin, has also shown antiamnesic [24] and antineurodegenerative [25] properties suggestive of its use in alleviating cognitive disorders.

While these individual reports in animal neurodegenerative models suggest the beneficial use of active components isolated from* Scutellaria baicalensis* and* Acacia catechu*, to the best of our knowledge, there is no report for the combined use of two standardized extracts in maintaining cognitive health. Acknowledging the complexity of aging process as well as global inflammation and generation of oxidative stress in relation to cognitive impairment, we believe that combining active fractions from these two medicinal plants together could be a preferred approach for mitigation of cognitive decline in healthy individuals. In summary, animal and human clinical results with UP326 suggest that there is significant attenuation of memory deficits in animal models and increases in speed and accuracy of processing in healthy human subjects, respectively. These results may be due to a reduction of eicosanoid inflammation and ROS effects on NF*κ*B-induced inflammatory pathways as a result of administration of UP326. The pathologic lesions associated with AD develop many years before these clinical manifestations; however, early diagnosis is still a major challenge for the prevention of the disease. Therefore, it could be inferred that regular consumption of a bioflavonoid supplement containing UP326 starting at a relatively early age in life could possibly prevent or delay the clinical manifestations of dementia.
