# Corrigendum to “Clinical and Preclinical Cognitive Function Improvement after Oral Treatment of a Botanical Composition Composed of Extracts from *Scutellaria baicalensis* and *Acacia catechu*”

**DOI:** 10.1155/2017/3493086

**Published:** 2017-07-03

**Authors:** Mesfin Yimam, Bruce P. Burnett, Lidia Brownell, Qi Jia

**Affiliations:** ^1^Unigen, Inc., 3005 1st Ave., Seattle, WA 98121, USA; ^2^Entera Health, Inc., 2000 Regency Parkway, Ste. 200, Cary, NC 27518, USA

In the article titled “Clinical and Preclinical Cognitive Function Improvement after Oral Treatment of a Botanical Composition Composed of Extracts from *Scutellaria baicalensis* and *Acacia catechu*” [1], there is an error in a sentence in the abstract section. The sentence, “In a separate human clinical trial, test subjects orally given 300 mg of UP326 BID for 30 days showed marked improvement in speed and accuracy of processing complex information in computer tasks and reduced their standard deviation of performance compared to baseline and the placebo group” should be corrected to “In a separate human clinical trial, test subjects orally given 300 mg of UP326 per day for 30 days showed marked improvement in speed and accuracy of processing complex information in computer tasks and reduced their standard deviation of performance compared to baseline and the placebo group.”
